# Turkish validity and reliability study of type 2 diabetes stigma assessment scale

**DOI:** 10.3906/sag-2006-255

**Published:** 2021-06-28

**Authors:** Bahar İNKAYA, Ezgi KARADAĞ

**Affiliations:** 1 Department of Nursing, Faculty of Health Science, Ankara Yıldırım Beyazıt University, Turkey Ankara; 2 Department of Oncology Nursing, Faculty of Nursing, Dokuz Eylül University, Turkey İzmir

**Keywords:** Type 2 diabetes, nursing, stigma, validity

## Abstract

**Background/aim:**

Stigma has a high incidence and adversely affects people with diabetes. In this context, patients face difficulties such as fear of losing their jobs, travel restrictions, isolation from social life, problems related to mental health, and feeling of wellness. The aim of this study was to investigate the validity and reliability of the stigma assessment scale in individuals with type 2 diabetes.

**Materials and methods:**

The study sample consisted of 153 diabetic individuals. The validity of language, content, and construct were examined to evaluate the validity of the type 2 diabetes stigma assessment scale. Cronbach’s alpha was used to assess internal consistency reliability.

**Results:**

The content validity index of 19 items which were detected as significant was found to be 0.86. The Cronbach’s alpha coefficient of the scale is 0.92. The results of the item analysis show that all factor loads are significant (t-value > ±1.96). The coefficient of correlation between type 2 diabetes stigma assessment scale and test–retest technique was 0.82.

**Conclusion:**

It was concluded that the stigma assessment scale is a valid and reliable measurement tool in individuals with type 2 diabetes mellitus. Nurses may use this tool to better understand and help relieve the prevalence and severity stigma of individuals with type 2 diabetes in Turkey.

## 1. Introduction

Diabetes mellitus (DM) is a chronic disease that requires complex continuous medical care, in which many risk factors can be controlled through glycemic control. According to the 2019 data of the International Diabetes Federation, there are 463 million people with diabetes worldwide. Turkey has the highest prevalence of diabetes among European countries and is predicted to be among the top 10 countries with the highest number (11.2 million) of people with diabetes in 2045 [1]. The results of the TURDEP-II study conducted in 15 provinces in Turkey identified that the prevalence of diabetes reached 13.7% in the Turkish adult population [2].

 In Turkish, the word ‘stigma’ means wound, black mark, indignity, disgrace and labelling. Prejudice, which is the basis of stigma [3,4], is a premature judgment of a person, object, or subject without an investigation. Judgment can be either positive or negative, and people’s behaviors are affected by prejudice. Ultimately, stigma resulting from prejudices leads to the exclusion and discrimination of people. Discrimination is the deprivation of certain rights and interests of people or groups in society due to stigmatization and prejudices. There are two types of stigma: imposed stigma is the actual rejection experience based on the inability of healthy/unhealthy individuals to be accepted into the social community. Perceived (internalized) stigma refers to the shame of healthy/unhealthy individuals about having stigmatized disease and the fear of being exposed to stigmatization [5–7].

Stigma in the Turkish society with type 2 diabetes is a concept that is ignored and not emphasized. Age, sex, education, occupation, marital status, social class, culture, religious beliefs, information about the disease, contact with mental illness, psychological type, and mass media are all factors affecting stigma. Studies have investigated the relationship between stigma and tuberculosis, obesity, epilepsy, and many mental chronic diseases [8–10]. Diabetes is a chronic disease that is very common in the community. Stigma has a high incidence and adversely affects people with diabetes. In a study, it was determined that diabetic patients with stigma had poor self-management and blood glucose control [11]. The stigma experienced by patients with type 2 diabetes affects their sense of self-worth, their attitude toward social participation, and self-management [12]. In addition, patients with type 2 diabetes who received intensive insulin therapy were found to have high stigma experiences [13].

Browne et al. [14] recognized this condition and evaluated the stigma with type 2 diabetes stigma assessment scale (DSAS-2) developed specifically for diabetes. Therefore, we aimed to adapt this scale because there is no measurement tool to measure such a phenomenon in Turkish society. 

## 2. Materials and methods

### 2.1. Design and study sample 

This study sought to characterize the validity and reliability of the type 2 diabetes stigma assessment scale, which was developed by Browne et al. [14], for Turkish society. Permission was obtained from the relevant author via e-mail, and a research protocol was established according to the author’s wishes. Data was collected from January 2018 to June 2018. 

### 2.2. Statistical analysis

The data of this study were analyzed using SPSS Statistics version 21.0 (IBM, Armonk, NY, USA). Sociodemographic and clinical characteristics were described as frequencies, means ± standard deviations.

The distributive normality of the variables was investigated by the Shapiro–Wilk test because of the number of units. The convenience of a scale for factor analysis and the suitability of the data are evaluated by Kaiser–Meyer–Olkin (KMO) and Bartlett tests. K-20 coefficients were used to investigate the reliability of the scales. The internal consistency of the scale was measured by Cronbach’s alpha and test–retest reliability was measured using intraclass correlation coefficient. Spearman’s correlation coefficient was used to investigate the relationships between the variables not normally distributed. The scales were evaluated with Pearson’s correlation analysis using the concurrent validity method. A level of p < 0.05 was taken to indicate significance. 

### 2.3. Data form

An introductory form for diabetic individuals consisted of questions about sex, age, educational status, occupation, economic status, and the year of diagnosis of diabetes.

#### 2.3.1. The type 2 diabetes mellitus stigma assessment scale

The scale was developed by Browne et al. [14] and consists of three dimensions, including different behaviors (6 items), blame and judgment (7 items), and self-stigmatization (6 items), with a total of 19 items. It is a 5-point Likert-type scale that is scored according to the selection between “strongly disagree” and “absolutely agree”. It consists of three dimensions, including different behaviors (items 1, 4, 7, 10, 14, 17 and possible range, 6–30), blame and judgment (items 2, 3, 5, 8, 12, 16, 19 and possible range, 7–35) and self-stigmatization (items 6, 9, 11, 13, 15, 18 and possible range, 6–30). The total stigma score of the scale is between 19 and 95 points. Higher scores correspond to higher levels of stigmatization [14]. 

#### 2.3.2. The Rosenberg self-esteem scale

The Rosenberg self-esteem scale (RSES) was developed in 1965 by Rosenberg. The validity and reliability study of the Turkish version was carried out by Cuhadaroglu in 1986, and the Cronbach’s alpha internal consistency coefficient was found to be 0.71. In this study, Cronbach’s alpha internal consistency coefficient of the Rosenberg self-esteem scale was found to be 0.77. The scale consists of 12 subscales and 63 items. The section, which is a Likert-type assessment scale, consists of 10 items. According to the internal assessment system of the scale, subjects receive scores between 0 and 6 points. The total score obtained from the scale shows high self-esteem from 0 to 1, moderate self-esteem from 2 to 4, and low self-esteem from 5 to 6. Higher scores correspond to lower levels of self-esteem Cuhadaroglu F. Self esteem in adolescents. Hacettepe University Faculty of Medicine, Specialty Thesis, Ankara, 1986.. The Rosenberg self-esteem scale is used for concurrent validity. The validity stages and translation of the scale consisted of the following steps.

### 2.4. Translation

In the first phase, three individuals from the translation team translated the scale from English to Turkish. The two experts whose native language is English and who can speak Turkish then back-translated the scale from Turkish into English. Finally, two Turkish language experts evaluated the compatibility of this scale with the Turkish language, and the comprehensibility of the scale was pretested.

### 2.5. Content validity

For the validity of the scale, expert opinions were consulted for the items of the scale and the items were evaluated according to the Lawshe technique. Factor analysis was used for the analysis of the structure validity. Factor analysis in behavioral sciences is applied in order to reveal structure(s) covered by the items of the scale. These structures are defined as the factors of the scale. The convenience of a scale for factor analysis and the suitability of the data are evaluated using the Kaiser–Meyer–Olkin (KMO) and Bartlett tests. The KMO test determines whether the distribution is sufficient for factor analysis, and results from 0.80 to 0.90 are considered very satisfactory. Bartlett’s test determines whether the hypothesis that the correlation matrix is equal to the unit matrix is accepted or rejected. The rejection of the hypothesis means that the correlation coefficient among the variables is different from 1.00 and that the measured variable is multivariate for the universe parameter. In order to determine the content validity index of the type 2 DM stigma assessment scale, expert opinions were consulted. By assessing the views from a total of 12 experts, the content validity ratio (CVR) was calculated for each item. Subsequently, the content validity index (CVI) was determined by calculating the mean of the calculated CVRs. This index is used to determine the decision of experts on the necessity of each item. This value is calculated for the level of eligibility of the items. Since there were twelve experts, it was concluded that items with a CVR value greater than 0.56 met the content validity [15–18]. After the calculation of the CVRs, it was determined that all items of the scale were considered eligible by experts. The content validity index of 19 items that were statistically significant was found to be 0.86. In addition, the result of the Kaiser–Meyer–Olkin (KMO) test for sampling adequacy was found to be 0.911 (>0.60) for the established diabetes scale. 

### 2.6. Concurrent validity 

For criterion validity, the relationship of scale scores with one or more external criteria is analyzed. This method can be applied as either concurrent (convergent validity, compliance validity, current validity) validity or predictive validity. In concurrent validity, the relationship with, if any, a previously developed scale measuring the same conceptual structure or, if not, with a scale developed using different scales measuring similar or related concepts is examined [15,19].

There is no scale that measures the concept of stigma in patients with type 2 diabetes in Turkey. Therefore, the nearest RSES scale was used. In our study, the relationship between the DSAS-2 and the RSES was evaluated with Pearson’s correlation analysis using the concurrent validity method. There was a statistically significant relationship between the total scores of the RSES and stigma scales (p < 0.05). This relationship was found to be weak and positive ( *r* = 0.337). Increases in the Rosenberg scale corresponded with increases in the stigma scale (Table 1). 

**Table 1:** Correlation test results for relationship between Rosenberg self-esteem scale and stigma scale scores.

Scales	Correlations				
		Treated differently	Blame and judgement	Self-stigma	Total DSAS-2
Blame and judgement	r	0.766**			
	p	0.001			
Self-stigma	r	0.721**	0.768**		
	p	0.001	0.001		
Total DSAS-2	r	0.889**	0.938**	0.903**	
	p	0.001	0.001	0.001	
Rosenberg	r	0.211**	0.321**	0.406**	0.337**
	p	0.001	0.001	0.001	0.001

### 2.7. Test–retest reliability 

In the item analysis, test–retest reliability means that a scale gives consistent results between two applications; that is, the scale shows invariance over time [18]. The DSAS-2 was performed 10 days after the first application, and the test–retest correlation was 0.82 (p ≤ 0.001). Item 5 was found to have the lowest test–retest correlation, whereas item 14 was found to have the highest correlation (Table 2). 

**Table 2:** Test–retest correlations.

Item	Factor	Test–retest correlation*
1. Bazı insanlar, tip 2 diyabetim olduğu için sorumluluklarımı (örn. iş, aile) yerine getiremeyeceğimi düşünüyorlar.	Treated differently	0.56
2. Sağlık profesyonelleri, tip 2 diyabetim olduğu için benimle ilgili olumsuz yargılarda bulunuyorlar.	Blame and judgement	0.64
3. Bazı insanlar, tip 2 diyabetim olduğu için şimdi veya geçmişte fazla kilolu olmam gerektiğini varsayıyorlar.	Blame and judgement	0.47
4. Bazı insanlar, tip 2 diyabetim olduğu için bana “hasta” veya “rahatsız”mışım gibi davranıyorlar.	Treated differently	0.58
5. Tip 2 diyabeti olan bireylerin etrafında suçlama ve utanç var.	Blame and judgement	0.34
6. Tip 2 diyabetim olduğu için, kendimi mahcup hissediyorum.	Self-stigma	0.62
7. Tip 2 diyabetim olduğu için ayrımcılığa maruz kaldım.	Treated diffrently	0.56
8. Sağlık profesyonelleri, tip 2 diyabeti olan kişilerin kendilerine bakamayacağını düşünüyor.	Blame and judgement	0.59
9. Tip 2 diyabetim olduğu için utanıyorum.	Self-stigma	0.45
10. Bazı insanlar, tip 2 diyabetim olduğu için beni daha az değerli buluyorlar.	Treated diffrently	0.63
11. Tip 2 diyabetim olduğu için, kendimi yetersiz hissediyorum.	Self-stigma	0.55
12. Tip 2 diyabetin, bir ‘yaşam tarzı” hastalığı olduğu yönünde olumsuz bir etiketleme var.	Blame and judgement	0.49
13. Tip 2 diyabetli olmak, bana başarısızmışım gibi hissettiriyor.	Self-stigma	0.65
14. Bazı insanlar yiyecek/içecek içeren sosyal birlikteliklerden uzak durmam gerektiğini düşündükleri için beni dışlıyorlar.	Treated differently	0.72
15. Tip 2 diyabetim olduğu için kendimi suçlu hissediyorum.	Self-stigma	0.68
16. Tip 2 diyabetim olmasının benim kendi hatam olduğu söylendi.	Blame and judgement	0.64
17. Tip 2 diyabetim olduğu için başkaları tarafından (örn. arkadaşlarım, iş arkadaşlarım, özel ilişkim) reddedildim.	Treated differently	0.62
18. Tip 2 diyabetim olduğu için kendimi suçluyorum.	Self-stigma	0.57
19. Tip 2 diyabetim olduğu için, bazı insanlar yemek seçimlerimi eleştiriyorlar.	Blame and judgement	0.59

* Spearman correlation test

There was a statistically significant relationship between scores of the different behaviors and the blame and judgment subscales (p < 0.05), which was found to be strong and positive (r = 0.766); that is, as the score of the different behaviors’ subdimension increases, the score of the blame and judgment subdimension will also increase. In addition, there was a statistically significant relationship between scores of subscales of the self-stigmatization and the different behaviors (p < 0.05), which was found to be moderately strong and positive (r = 0.721). There was also a statistically significant relationship between scores of the self-stigmatization and the blame and judgment subscales (p < 0.05), which was found to be strong and positive (r = 0.768); that is, as the score of the self-stigmatization subdimension increases, the score of the blame and judgment subdimension will also increase. The scores of subscales of the stigma and the different behaviors were also strongly and positively related (p < 0.05; r = 0.7889); that is, higher total scores of the dimensions of the stigma scale correspond with higher scores of the different behaviors’ subdimension. There was a statistically significant relationship between the total scores of the stigma scale dimensions and the score of the blame and judgment subdimension (p < 0.05), and this relationship was also found to be very strong and positive (r = 0.938). As the score of the stigma scale increases, the score of the blame and judgment subdimension will also increase. There was a statistically significant relationship between total scores of the dimensions of the stigma scale and the self-stigmatization subscale (p < 0.05). This relationship was found to be very strong and positive (r = 0.903) (Table 1). As the total scores of the dimensions of the stigma scale increases, the self-stigma subscale scores also increase. 

### 2.8. Ethical considerations 

The ethical approval of this study was obtained from the Ethical Committee for Clinical Trials of Ankara Yıldırım Beyazıt University (Protocol number: 2017/39). Before the study, written informed consent was obtained from all patients with type 2 diabetes mellitus. 

## 3. Results

### 3.1. Sample characteristics

The study population consisted of type 2 diabetes mellitus patients between 18 and 75 years of age who were admitted to a training and research hospital in Ankara and who had no communication problems and were able to speak and understand Turkish. The data of the study were collected between March and September 2018. The number of patients in the sample should be 5 to 10 times the number of items in the validity and reliability studies [15]. Therefore, it was planned to include 200 patients for DSAS-2 with 19 items. The sample of the study consisted of 153 patients with type 2 diabetes who agreed to participate in the study. Of the diabetic subjects who participated in this study, 55.19% were female. The rate of patients with 16 or more years of diagnosis was 20.13%. Among these individuals, 49.35% were primary school graduates, and 40.26% were housewives. The mean HbA1c levels of the sample were 7.34 ± 2.11. 

### 3.2. Construct validity

After providing the language and content validities of the scale, confirmatory factor analysis was performed to determine construct validity and whether an overlap was detected between the original structure of the scale and the factor structures. The factor structure of the scale is shown in Figure. 

**Figure:**
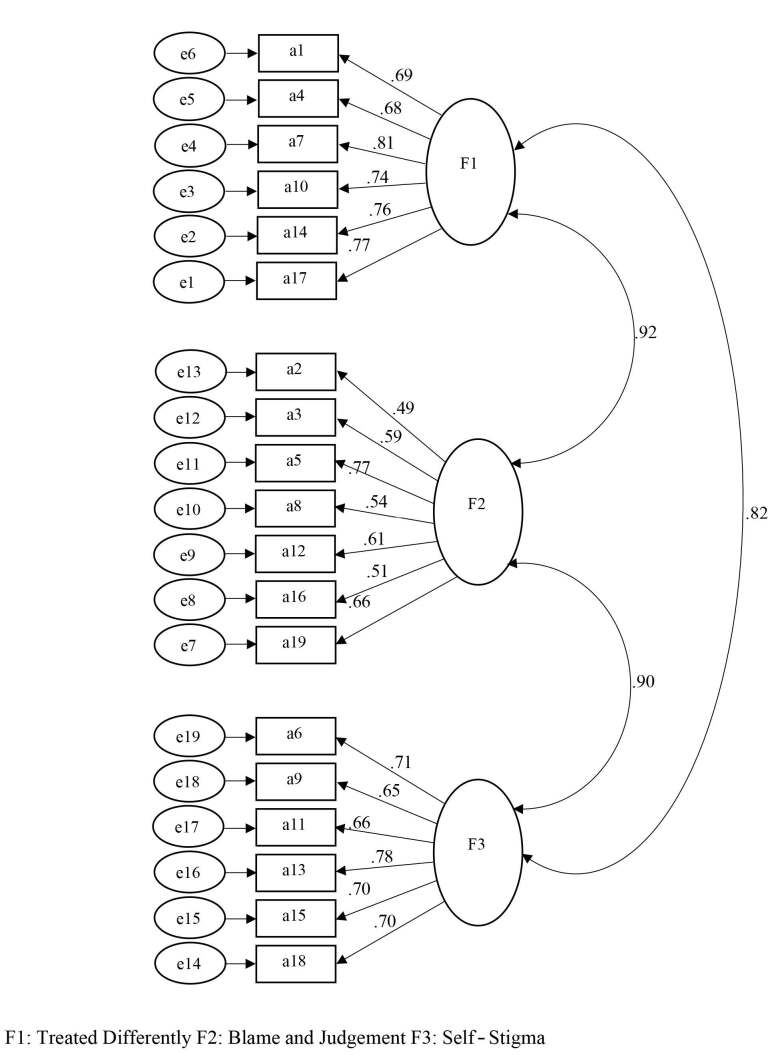
Path diagram of DSAS-2.

The compliance indices were found as *X* *2* */sd* = 1.871, *RMR* = 0.055, *RMSEA* = 0.075, *CFI* = 0.971, and *GFI* = 0.96, respectively [15–18]. An analysis of the coefficients showing the relationship between the observed variables of the model representing the factorial structure showed that all coefficients were adequate. Considering the compliance statistics calculated by CFA, the previously determined structure of the scale adapts highly to the collected data. The results obtained from the analysis show that the factor structure is generally within acceptable limits (Figure). This situation shows that the model determined theoretically in Figure corresponds to the sample data. The analysis of the significance test results for the path coefficients given in Table 3 shows that all factor loads are significant ( *t-* value > ±1.96). In addition, according to standardized parameter estimations, it is concluded that all indicators are in conformity with the relevant structure and are of the correct mark and size. All estimates for coefficients were significant (p < 0.05). 

**Table 3:** Regression and t-values of DSAS-2.

Factor	İtems	Regression values	t-values
Treated differently Treated differentlyTreated differentlyTreated differentlyTreated differentlyTreated differently	a17	0.779	
	a14	0.751	9.863
	a10	0.736	9.599
	a7	0.806	10.748
	a4	0.669	8.596
	a1	0.691	8.922
Blame and judgement Blame and judgementBlame and judgementBlame and judgementBlame and judgementBlame and judgementBlame and judgement	a19	0.558	5.592
	a16	0.425	4.62
	a12	0.576	5.679
	a8	0.543	5.448
	a5	0.797	6.972
	a3	0.59	5.775
	a2	0.492	5.061
Self-stigma Self-stigmaSelf-stigmaSelf-stigmaSelf-stigmaSelf-stigma	a18	0.589	7.760
	a15	0.6	9.07
	a13	0.852	7.846
	a11	0.681	7.03
	a9	0.738	7.093
	a6	0.72	7.374

### 3.3. Internal consistency reliability

For the reliability analysis of the scale, Cronbach’s alpha analysis (internal consistency analysis), an item analysis (item-total correlation, corrected item-total correlation and item discrimination indexes) and a test–retest technique were used. Cronbach’s alpha analysis assesses whether the items on the scale are consistent with each other and whether they measure the same characteristic. In other words, Cronbach’s alpha analysis is a measure of the internal consistency and homogeneity of the items on the scale (higher Cronbach’s alpha coefficients correspond with more reliable scales) [15,18]. The analysis of the reliability dimension of the scale showed that Cronbach’s alpha coefficient for the reliability of items of the different behaviors subscale was 0.87. Cronbach’s alpha coefficient for the reliability of items of the blame and judgment subscale was 0.78, and for the reliability of items of the self-stigmatization subscale, it was 0.85. In addition, Cronbach’s alpha coefficient for the reliability of items of the stigma scale was 0.92. A coefficient greater than 0.70 indicates that the scale is reliable. Cronbach’s alpha coefficient for the reliability of items of the Rosenberg scale was 0.72. Note that coefficient values greater than 0.70 indicate that the scale is reliable (Table 4).

**Table 4:** Cronbach’s alpha conclusions on the reliability of subscales.

	Cronbach’s alpha	n
Rosenberg self-esteem scale	0.72	10
Treated differently	0.87	6
Blame and judgement	0.777	7
Self-stigma	0.847	6
Total DSAS-2	0.927	19

## 4. Discussion

The present study investigated the validity and reliability of the stigma assessment scale for patients with type 2 diabetes in Turkish society, and the obtained results demonstrate a high level of validity and reliability. This is an important scale that is easily applicable in Turkish society and therefore needs to be considered in further research. In Turkey, there is a dominance of the traditional social structure. Therefore, individuals with chronic diseases (especially cancer, diabetes, AIDS, and TBC) can be perceived differently by society or individuals; thus, the perception of stigmatization may occur. Due to the absence of a scale for assessing the stigma associated with diabetes in Turkey, this study is of particular importance. 

In this study, confirmatory factor analysis (CFA) was used to evaluate the factor structure of the developed diabetes scale and to determine the items that were valid in the measurement model. The results obtained show that the factor structure is generally within acceptable limits. The results also show that the three-dimensional factorial structure of the scale provides adequate compliance values. These results support the results found in studies carried out at the stage of development of the original scale [13]. The three-factor structure, which corresponds to the original scale and includes the dimensions of ‘different behaviors’, ‘blame and judgment’ and ‘self-stigmatization’ has been confirmed. 

According to the analysis of the compliance indices, it was decided that the original structure of the scale was highly compatible with the collected data. This result can be regarded as evidence that the scale is eligible for Turkish society. 

 In our study, the relationship between the DSAS-2 and RSES scales was evaluated with Pearson’s correlation analysis using the concurrent validity method. There was a statistically significant positive relationship between total scores of the RSES scale and the DSAS-2 total scale score and subscale scores (p < 0.05). The same relationship was found in the study by Browne et al. [14]. In addition, many studies have found that stigmatization is strongly and consistently associated with negative psychological conditions such as depression, anxiety, anger, low self-esteem, and demoralization [20–23]. In a study on cancer patients, it was found that stigmatization decreased the quality of life of patients and negatively affected emotional functions [22]. In another study on people with diabetes, it was found that diabetes-related stigma has a significant effect on psychological distress, depressive symptoms, and self-esteem [24]. Similarly in another study, it was observed that people with diabetes tried to hide their diseases [25]. These results can be regarded as an indicator that stigma is as important as other psychosocial concepts.

In addition, Cronbach’s alpha coefficient for the reliability of items of the stigma scale was 0.93. In the original scale developed by Browne et al. [14], Cronbach’s alpha coefficient, which is a measure of the reliability of the scale, was found to be 0.95. These results, in analogy with the original scale, show that the DSAS-2-Turkey scale also has a high reliability value. In this study, Cronbach’s alpha coefficient of the scale and its subdimensions showed that they all have high levels of reliability: 0.87 for the different behavior subdimensions, 0.77 for the blame and judgment subdimension, and 0.85 for the self-stigmatization subdimension. Although similar results were obtained, Cronbach’s alpha coefficient of the blame and judgment and self-stigmatization subscales was significantly higher in the Browne et al.’s [14] study (0.90). 

In this study, the reliability coefficient of the test–retest test was found to be 0.86 (p = 0.001). Moreover, item 5 was found to have the lowest test–retest correlation, whereas item 14 was found to have the highest correlation. The low correlation coefficient of item 5 in the blame and judgment subdimension can be attributed to individual variations of the disease perception. The highest correlation coefficient of 14 items questioning the area of different behaviors can be attributed to tabooing and exaggerating nutrition by individuals with diabetes due to their disease and thus to the dominance of the idea of exclusion in social environments.

## 5. Conclusion

We conclude that the Turkish version of the DSAS-2, which is composed of 19 questions, is a valid and reliable scale. Stigma in type 2 diabetes patients is a new concept in Turkey, and no measurement tool is available for it. Diabetes is a chronic condition that places a significant emotional and social burden on the person living with it; yet, the social aspects of diabetes remain underresearched. Type 2 diabetes stigma assessment scale will enable nurses to become aware of the importance of stigma. With continual use of the DSAS-2, the degree of stigma will be accurately assessed. As a result of these assessments, it would be possible to provide patients with different treatment strategies and nursing care in addition to early intervention to help reduce stigma. Clinical nurses/diabetes nurses would support the patients whose stigma levels were determined. With continual use of the DSAS-2 in diabetic patients, the degree of stigma will be accurately assessed.

However, further work is needed to provide psychological support for stigma, to plan appropriate training programs, and to aid participation of family members in these trainings to provide further support to diabetic patients. Finally, we recommend the use of DSAS-2 for carrying out cross-cultural research that evaluates stigma.
